# Prior antiplatelet therapy in patients undergoing endovascular treatment for acute ischemic stroke: Results from the MR CLEAN Registry

**DOI:** 10.1177/1747493020946975

**Published:** 2020-08-14

**Authors:** Rob A van de Graaf, Sanne M Zinkstok, Vicky Chalos, Robert-Jan B Goldhoorn, Charles BLM Majoie, Robert J van Oostenbrugge, Aad van der Lugt, Diederik WJ Dippel, Yvo BWEM Roos, Hester F Lingsma, Adriaan CGM van Es, Bob Roozenbeek, on behalf of the MR CLEAN Registry investigators

**Affiliations:** 1 Department of Neurology, 6993 Erasmus MC University Medical Center, Rotterdam, The Netherlands; 2 Department of Radiology and Nuclear Medicine, 6993 Erasmus MC University Medical Center, Rotterdam, The Netherlands; 3 Department of Neurology, 3913 Tergooi, Hilversum, The Netherlands; 4 Department of Public Health, 6993 Erasmus MC University Medical Center, Rotterdam, The Netherlands; 5 Department of Neurology, Cardiovascular Research Institute Maastricht, Maastricht University Medical Center, Maastricht, The Netherlands; 6 Department of Radiology and Nuclear Medicine, Amsterdam UMC, Location AMC, Amsterdam, The Netherlands; 7 Department of Neurology, Amsterdam UMC, University of Amsterdam, Location AMC, Amsterdam, The Netherlands

**Keywords:** Antiplatelet, endovascular, MR CLEAN Registry, thrombectomy, stroke

## Abstract

**Background:**

Antiplatelet therapy may increase the risk of symptomatic intracranial hemorrhage after endovascular treatment for ischemic stroke but may also have a beneficial effect on functional outcome. The aim of this study is to compare safety and efficacy outcomes after endovascular treatment in patients with and without prior antiplatelet therapy.

**Methods:**

We analyzed patients registered in the MR CLEAN Registry between March 2014 and November 2017, for whom data on antiplatelet therapy were available. We used propensity score nearest-neighbor matching with replacement to balance the probability of receiving prior antiplatelet therapy between the prior antiplatelet therapy and no prior antiplatelet therapy group and adjusted for baseline prognostic factors to compare these groups. Primary outcome was symptomatic intracranial hemorrhage. Secondary outcomes were 90-day functional outcome (modified Rankin Scale), successful reperfusion (extended thrombolysis in cerebral infarction score ≥2B) and 90-day mortality.

**Results:**

Thirty percent (*n* = 937) of the 3154 patients were on prior antiplatelet therapy, who were matched to 477 patients not on prior antiplatelet therapy. Symptomatic intracranial hemorrhage occurred in 74/937 (7.9%) patients on prior antiplatelet therapy and in 27/477 (5.6%) patients without prior antiplatelet therapy adjusted odds ratio 1.47, 95% confidence interval 0.86–2.49. No associations were found between prior antiplatelet therapy and functional outcome (adjusted common odds ratio 0.87, 95% confidence interval 0.65–1.16), successful reperfusion (adjusted odds ratio 1.23, 95% confidence interval 0.77–1.97), or 90-day mortality (adjusted odds ratio 1.15, 95% confidence interval 0.86–1.54).

**Conclusion:**

We found no evidence of an association of prior antiplatelet therapy with the risk of symptomatic intracranial hemorrhage after endovascular treatment, nor on functional outcome, reperfusion, or mortality. A substantial beneficial or detrimental effect of antiplatelet therapy on clinical outcome cannot be excluded. A randomized clinical trial comparing antiplatelet therapy versus no antiplatelet therapy is needed.

## Introduction

Approximately 50% of patients with ischemic stroke do not recover to functional independence after endovascular treatment (EVT).^1^ Although pre-stroke disability and large baseline infarct core are known causes of these poor outcomes, incomplete microvascular reperfusion—a potentially reversible process—might contribute to these poor outcomes as well. One of the causes of incomplete microvascular reperfusion is the formation of microthrombi occluding the distal capillary bed. These microthrombi are abundantly present after focal cerebral ischemia in the distal vascular territory. The formation of microthrombi might be promoted by vessel wall damage caused by EVT.^2,3^ Use of antiplatelet drugs could potentially reduce periprocedural formation of microthrombi by inhibiting platelet aggregation and inflammation of the vessel wall, which could ultimately improve microvascular reperfusion.^3^ On the other hand, one randomized trial showed that antiplatelet therapy increases the risk of symptomatic intracranial hemorrhage (sICH) when administered early—within 90 min—after intravenous treatment with alteplase.^4^ However, this trial did not focus on the subpopulation of patients with ischemic stroke caused by a large vessel occlusion undergoing EVT. In these patients, the beneficial effect of platelet inhibition could counterbalance the detrimental effects of increase risk of sICH. As interventionists are familiar with periprocedural use of antiplatelet agents during non-stroke neurovascular procedures (i.e. stenting), this treatment might be an easily applicable therapy of adjunctive value in EVT for stroke. However, as antiplatelet agents are not administered systematically during EVT for acute ischemic stroke and current evidence is limited to small observational studies investigating the association of prior antiplatelet therapy with sICH risk and functional outcomes, there are conflicting results.^5^ The evaluation of risks and benefits of prior antiplatelet therapy in a large cohort of patients treated with EVT could provide useful information for clinical practice. The aim of this study is to compare safety and efficacy outcomes, after EVT of patients with and without prior antiplatelet therapy.

## Methods

### Study design

We used data from the MR CLEAN Registry that is a prospective national multicenter study including all consecutive patients treated with EVT for ischemic stroke in the Netherlands. The complete methods and definition of variables of the MR CLEAN Registry have been described elsewhere.^6^ For the present study, we selected patients who were registered between March 2014 and November 2017 and adhered to the following criteria: age 18 years or older; treatment in a center that participated in the MR CLEAN trial; presence of a proximal intracranial occlusion in the anterior circulation confirmed on computed tomography angiography (CTA) (intracranial carotid artery (ICA), intracranial carotid artery terminus (ICA-T), middle cerebral artery (M1/M2), or anterior cerebral artery (A1/A2)); and groin puncture within 6.5 h after symptom onset and known data on prior antiplatelet therapy. The current observational study was guided by the STROBE statement.^7^

### Ethical considerations and data availability

The central medical ethics committee of the Erasmus MC, University Medical Center Rotterdam, the Netherlands, evaluated the study protocol and granted permission to carry out the study as a registry (MEC-2014-235). This approval extends to all participating centers in the Netherlands. Coded data were obtained and stored at Erasmus MC, and scientific analyses were approved and supervised by a central writing committee. The MR CLEAN Registry study protocol is available on http://www.mrclean-trial.org/docs/latestprotocol.pdf. Data cannot be made available, as no patient approval has been obtained for sharing coded data. However, syntax files and output of statistical analyses (R 3.5.0) will be made available upon request.

### Prior antiplatelet therapy

Prior antiplatelet therapy was defined as the use of any antiplatelet agent at baseline, reported by the local investigators. We compared outcomes of patients that were on antiplatelet therapy prior to the EVT procedure to patients not on antiplatelet therapy. Data on which specific antiplatelet agent was used before EVT were not prospectively collected in the MR CLEAN Registry. For insight into dual antiplatelet usage, we retrospectively evaluated all available patient discharge letters on this specific issue. Acute administration of any antiplatelet agents during EVT is not part of common practice in the Netherlands. At the discretion of the treating physician, acute administration of antiplatelets was possible in those patients requiring immediate carotid artery stenting; this concerns only a few patients and was not recorded in this registry. Patients with non-cardioembolic ischemic stroke received antithrombotic medication according to local guidelines (either clopidogrel or acetylsalicylic acid, with appropriate loading dose). In patients who also received intravenous thrombolytics, antiplatelet therapy was delayed until >24 h after stroke thrombolysis.

### Outcome measures

The primary outcome was the occurrence of sICH, before final follow-up assessment at 90 days, defined as neurological deterioration (increase of four points or more on the National Institutes of Health Stroke Scale (NIHSS)) and a compatible cerebral hemorrhage seen on imaging assessed by an independent imaging core laboratory.

Secondary outcomes were functional outcomes at 90 days (range ± 14 days) on the modified Rankin Scale (mRS), which is a seven-point ordinal scale ranging from 0 “no symptoms” to 6 “dead” (both ordinal and dichotomized for functional independence (0–2 vs. 3–6),^8^ successful reperfusion of the distal macrovascular territory (extended Thrombolysis In Cerebral Infarction grade ≥2B) at the end of the EVT assessed by an independent imaging core laboratory, NIHSS score 24–48 h after intervention, and within 90 days occurrence of mortality, progression of ischemic stroke, new ischemic stroke, extracranial hemorrhage, and cardiac ischemia.

### Statistical methods

Differences in baseline characteristics were assessed for both categorical and dichotomous variables using *χ*^2^ test for categorical variables, independent samples *t*-test for normally distributed continuous variables, and Kruskal–Wallis for non-parametric testing. Any mRS score (except for occurrence of death) assessed within 30 days of symptom onset was considered invalid and treated as missing. We assume “missing” in any (both safety and efficacy) outcome assessment to be distributed at random. For the purpose of unbiased estimation of associations of outcome with baseline characteristics, we used multiple imputation by chained equations and pooled data over five imputed datasets.^9,10^ All baseline data and outcomes that are reported are crude and not imputed. A description of the exact imputation settings used is provided in Suppl. File 1. To reduce possible confounding by indication, we performed propensity-score matching, using a within approach, performing propensity-score matching within each imputed dataset averaging the effect estimates.^11^ The propensity score for each individual was defined as the probability of being on the treatment (prior antiplatelet therapy) given the patient’s baseline characteristics and comorbidities. Variables used to retrieve the propensity score were required to be factors potentially related to the choice of treatment assignment remaining inclusive. In case of uncertainty whether a variable was related to treatment assignment advantage was given to this variable. Subsequently we performed nearest-neighbor matching on the derived propensity score with replacement setting a caliper of 0.25 SD of the logit for propensity score.^12,13^ To assess whether the propensity-score model has been adequately specified, we evaluated baseline characteristics distributions before and after matching and evaluated propensity-score densities graphically.^14^ Within the propensity-score-matched cohort, we performed binary and ordinal logistic and linear regression analyses as appropriate and additionally adjusted for important prognostic covariates to optimally reduce residual imbalances in observed covariates between patients with and without prior antiplatelet therapy.^15,16^ The selection of covariates was based on prior knowledge and included NIHSS at admission, treatment with intravenous alteplase, location of the intracranial occlusion, Alberta Stroke Program Early Computed Tomography Score (ASPECTS) at baseline, CTA collateral grade at baseline, and time from onset to reperfusion. Sensitivity analyses using binary and ordinal and linear logistic regression for the full cohort (without propensity-score matching) were performed. Additional subgroup analyses on associations of antiplatelet therapy were performed for history of myocardial infarction, history of prior stroke, treatment with both intravenous alteplase and EVT, treatment with EVT only, successful reperfusion, and for prior oral anticoagulant use. Associations are presented as (adjusted common) odds ratio (a(c)OR) with 95% confidence interval (CI). Because there is consensus on the ordering of the outcome scale in this case (each score on the mRS is more favorable than a one point lower score), the common OR can be presented and interpreted as a summary estimate of the treatment effect, even if the underlying proportional odds assumption would be violated.^17^ Therefore, we decided not to formally test this assumption. All statistical analyses were performed with R version 3.5.0 (R foundation for Statistical Computing, Vienna, Austria).

## Results

### Study population

**Figure 1.**
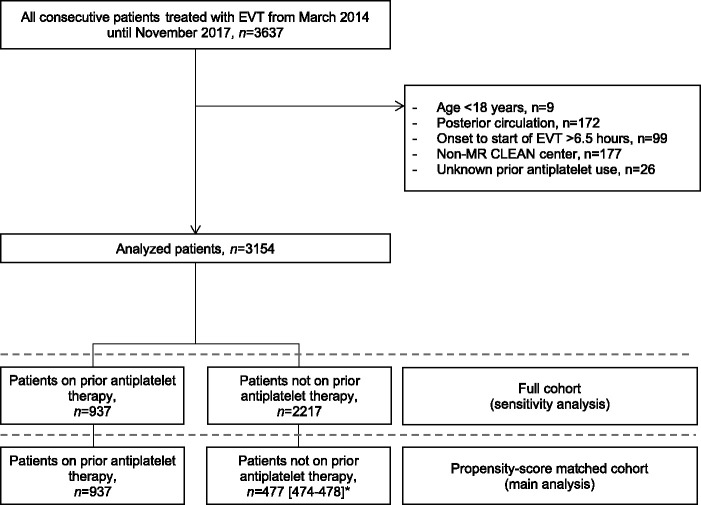
Flowchart. EVT, endovascular treatment; MR CLEAN, Multicenter randomized clinical trial of endovascular treatment of acute ischemic stroke.

A total of 3154 patients were analyzed, of which 937 patients (30%) were on prior antiplatelet therapy (Figure 1). Based on the retrospective discharge letter evaluation, data on the specific antiplatelet agent used were missing in 360 of the 937 patients. Among patients with available data, 83% (480/577) were on a single antiplatelet therapy and 17% on dual antiplatelet therapy (97/577). Patients on dual antiplatelets used a combination of acetylsalicylic acid and dipyridamole in 51% of the cases (50/97) and a combination of acetylsalicylic acid and clopidogrel in 41% of the cases (40/97). Patients on prior antiplatelet therapy were older, were more often male, had lower international normalized ratios, had more comorbidities (i.e. history of ischemic stroke, atrial fibrillation, hypertension, diabetes, and myocardial infarction), and had higher pre-stroke mRS scores (Table 1). Also, these patients were more often using other types of medication, were more often eligible for intravenous alteplase, and differed from patients not on prior antiplatelet therapy in baseline imaging characteristics (i.e. occlusion location, ASPECTS, and collateral filling).

**Table 1.** Baseline demographics before propensity-score matching [full cohort] and after matching [propensity-score-matched cohort].

	Propensity-score-matched cohort		Full cohort		
	Prior APT (*n* = 937)^a^	No prior APT (*n* = 477)^a^	Prior APT (*n* = 937)	No prior APT (*n* = 2217)	Missing
*Common patient characteristics*					
Age	74 (12)	73 (13)	74 (12)	69 (15)	0
Male sex	521 (56)	259 (54)	521 (56)	1121 (51)	0
NIHSS at baseline	16 [11, 20]	16 [12, 20]	16 [11, 20]	16 [11, 19]	14/32
Ischemia in left hemisphere	506 (54)	246 (52)	501 (54)	1169 (53)	8/10
Systolic blood pressure	149 (25)	153 (24)	149 (25)	150 (25)	27/57
Diastolic blood pressure	80 (15)	83 (16)	80 (15)	83 (16)	27/65
INR	1.1 (0.3)	1.2 (0.4)	1.1 (0.3)	1.2 (0.4)	189/398
Glucose level (mmol/L)	7.5 (2.5)	7.6 (2.5)	7.6 (2.5)	7.3 (2.5)	118/239
Thrombocyte count (10^9^/L)	250 (97)	249 (84)	250 (99)	249 (82)	121/308
*Medical history*					
Previous stroke	325 (35)	103 (22)	322 (35)	204 (9.2)	9/11
Atrial fibrillation	180 (19)	125 (26)	177 (19)	575 (26)	17/17
Hypertension	632 (68)	301 (63)	615 (68)	1005 (46)	26/31
Diabetes mellitus	210 (23)	93 (20)	210 (23)	291 (13)	3/14
Myocardial infarction	305 (33)	88 (18)	296 (33)	144 (6.6)	26/31
Peripheral arterial disease	171 (18)	66 (14)	166 (18)	124 (5.7)	25/31
Pre-stroke mRS > 2	149 (16)	67 (14)	139 (15)	221 (10)	37/32
*Medication use*					
DOAC	10 (1.1)	11 (2.3)	10 (1.1)	94 (4.3)	10/13
Coumarin	41 (4.4)	53 (11)	41 (4.4)	366 (16.6)	2/8
Blood pressure lowering medication	677 (72)	286 (60)	666 (72)	1018 (46.5)	14/27
Statin	598 (64)	158 (33)	583 (64)	515 (23.6)	19/32
*Imaging*					
Occluded segment					44/89
Intracranial ICA	38 (4.1)	25 (5.3)	36 (4.0)	119 (5.6)	
ICA-T	170 (18)	101 (21)	161 (18)	468 (22)	
M1	560 (60)	272 (57)	535 (60)	1218 (57)	
M2	157 (17)	75 (16)	151 (17)	310 (15)	
Other (e.g. M3, ACA)	11 (1.2)	3 (0.6)	10 (1.1)	13 (0.6)	
Reperfusion grade before intervention (eTICI)					48/119
0	753 (80)	377 (79)	717 (81)	1667 (80)	
1	47 (5.0)	30 (6.3)	44 (4.9)	141 (6.7)	
2 A	46 (4.9)	24 (5.0)	43 (4.8)	101 (4.8)	
2B	65 (7.0)	32 (6.7)	63 (7.1)	136 (6.5)	
2C	15 (1.4)	4 (0.8)	11 (1.2)	16 (0.8)	
3	11 (1.2)	10 (2.1)	11 (1.2)	37 (1.8)	
ASPECTS	9 [8, 10]	9 [7, 10]	9 [8, 10]	9 [7, 10]	33/70
ASPECTS ≤ 7	226 (24)	123 (26)	216 (24)	549 (26)	33/70
CTA collateral grade					60/141
Grade 0: Absent collaterals	50 (5.3)	32 (6.6)	48 (5.5)	136 (6.6)	
Grade 1: Occluded area filling <50%	377 (40)	166 (35)	354 (40)	708 (34)	
Grade 2: Occluded area filling >50% but <100%	335 (36)	190 (40)	312 (36)	832 (40)	
Grade 3: Occluded area filling 100%	175 (19)	88 (19)	163 (19)	400 (19)	
*Workflow (min)*					
Time from symptom onset to admission ER (intervention center)	135 [60, 186]	131 [65, 185]	136 [60, 187]	131 [63, 187]	56/100
Time from admission ER to groin puncture	61 [35, 92]	62 [38, 93]	59 [35, 89]	60.0 [35, 90]	107/180
Duration procedure	58 [39, 85]	59 [38, 85]	58 [39, 84]	59 [38, 83]	88/194
Time from symptom onset to recanalization	250 [201, 307]	250 [198, 314]	251 [202, 305]	250 [198, 312]	69/130
*Treatment*					
Treatment with intravenous alteplase	744 (79)	355 (75)	741 (79)	1668 (75)	3/5
General anesthetic management	218 (23)	115 (24)	204 (23)	549 (26)	59/134
Administration of intra-arterial thrombolytic	22 (2.3)	11 (2.3)	22 (2.3)	57 (2.6)	0
Periprocedural heparin administration	259 (28)	128 (27)	259 (28)	582 (26)	0

ACA, anterior cerebral artery; APT, antiplatelet therapy; ASPECTS, Alberta stroke program early CT score; DOAC, direct oral anticoagulant; ER, emergency room; eTICI, extended thrombolysis in cerebral infarction; ICA (T), intracranial carotid artery (terminus); M(*segment*), middle cerebral artery; mRS, modified Rankin Scale; NIHSS, National Institutes of Health Stroke Scale.

Note: Baseline variables of patients on prior antiplatelet therapy vs. no prior antiplatelet therapy. Continuous and ordinal data are presented as mean (SD) for normal distributed data or as median [IQR] for skewed data. Categorical data are presented as numbers (%).

a Calculated after handling missing data using multiple imputation procedures and performing propensity-score matching within the derived five imputation sets. Variables were averaged over five imputation sets. On average 477 patients (number of matches over five imputations ranged between 474 and 478) not on prior antiplatelets were matched to 937 patients who were on prior antiplatelet therapy (allowing replacement).

### Propensity-score matching

On average, 477 patients (number of matches over five imputations ranged between 474 and 478) not on prior antiplatelets were matched to 937 patients who were on prior antiplatelet therapy. In the propensity-score-matched cohort, baseline characteristics were more similar between groups compared to the full cohort suggesting that reasonable balance was obtained (Table 1). Also, visual balance check of the propensity score was reasonably improved when comparing the distributions before matching (full cohort) to those after matching (propensity-score-matched cohort; supplemental material II).

### Outcomes

**Figure 2.**
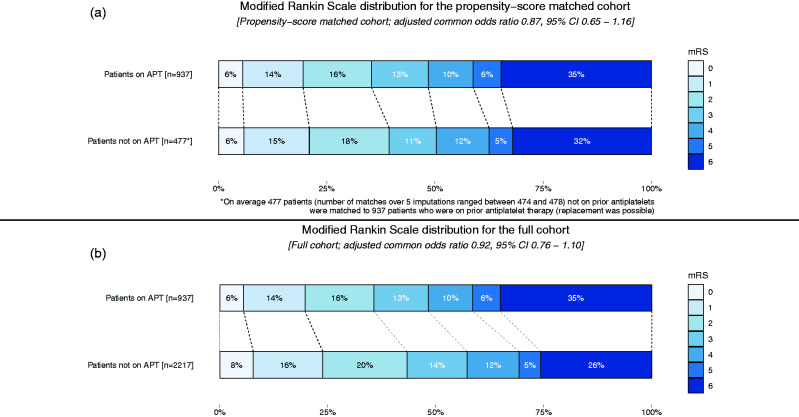
mRS distribution for the propensity-score-matched cohort (a) and full cohort (b) for patients on prior antiplatelet therapy and not on prior antiplatelet therapy. Missing values on mRS were handled by multiple imputation in 6.7% of patients. APT, antiplatelet therapy; CI, confidence interval; EVT, endovascular treatment; MR CLEAN, Multicenter randomized clinical trial of endovascular treatment of acute ischemic stroke; mRS, modified Rankin Scale.

In the propensity-score-matched cohort, no significant difference in sICH risk was observed between patients who were on prior antiplatelet therapy and those not on prior antiplatelet therapy (74/937 [7.9%] vs. 27/477 [5.6%]; aOR 1.47, 95% CI 0.86–2.49; Table 2). Also, no associations were found between prior antiplatelet therapy and functional outcome (median mRS 4 [IQR: 2–6] vs. 4 [2–6]; acOR 0.87, 95% CI 0.65–1.16; Figure 2), successful reperfusion (aOR 1.23, 95% CI 0.77–1.97), mortality (aOR 1.15, 95% CI 0.86–1.54), or the other secondary outcomes. In the sensitivity analysis, in the full cohort (without propensity-score matching), we found neither a difference in sICH risk (aOR 1.48, 95% CI 0.99–2.20) nor a difference in functional outcome (acOR 0.92, 95% CI 0.76–1.10; Figure 2) between groups. Only in the subgroup of patients with a prior stroke, we found that risk of sICH was increased for patients on prior antiplatelet therapy compared to those not on antiplatelet therapy (aOR 11.08, 95% CI 2.04–60.31). We did not detect a beneficial association on functional outcome of prior antiplatelet therapy in the subgroup analysis of patients with successful reperfusion (eTICI ≥ 2B). Results of the sensitivity analysis are presented together with additional subgroup analyses in the supplemental material III and IV.

**Table 2.** Primary and secondary outcomes in patients on prior antiplatelet therapy vs. no prior antiplatelet therapy in the propensity-score-matched cohort

	Prior APT (*n* = 937)^a^	No prior APT (*n* = 477)^a^	a(c)OR, (95% CI)^b^
*Primary outcome*			
Symptomatic intracranial hemorrhage	74 (7.9)	27 (5.6)	1.47 (0.86-2.49)
*Secondary outcomes*			
mRS at 90 days	4 [2, 6]	4 [2, 6]	0.87 (0.65–1.16)
mRS ≤ 2 at 90 days	334 (36)	180 (38)	0.88 (0.64–1.19)
NIHSS at 24–48 h	11 [4, 18]	11 [4,18]	0.14 (−0.72 to 1.01)^c^
Reperfusion grade after intervention (eTICI ≥ 2B)	587 (63)	273 (57)	1.23 (0.77–1.97)
Mortality within 90 days	329 (35)	157 (33)	1.15 (0.86–1.54)
Progression of stroke	71 (7.6)	49 (10)	0.70 (0.41–1.20)
New ischemic stroke	14 (1.5)	9 (2.0)	0.74 (0.28–1.93)
Extracranial hemorrhage	22 (2.3)	13 (2.8)	0.81 (0.37–1.82)
Cardiac ischemia	7 (0.7)	4 (0.8)	1.02 (0.19–5.43)

a(c)OR, adjusted (common) odds ratio; APT, antiplatelet therapy; CI, confidence interval; eTICI, extended thrombolysis in cerebral infarction; mRS, modified Rankin Scale.

Note: Primary and secondary outcomes of patients on prior antiplatelet therapy vs. no prior antiplatelet therapy. Skewed continuous and ordinal data are presented as median [IQR]. Binary data are presented as numbers (%).

a Calculated after handling missing data using multiple imputation procedures and performing propensity-score matching within the derived five imputation sets. Variables were averaged over five imputation sets. On average 477 patients (number of matches over five imputations ranged between 474 and 478) not on prior antiplatelets were matched to 937 patients who were on prior antiplatelet therapy (allowing replacement).

b *Variables used for retrieving propensity score and matching:* sex, age, pre-stroke disability (mRS), direct oral anticoagulant therapy, vitamin K antagonist therapy, previous stroke, myocardial infarction, peripheral artery disease, diabetes mellitus, hypertension, and atrial fibrillation; *variables in the additional adjustment model:* NIHSS at admission, intravenous alteplase, occlusion segment, ASPECTS at baseline, onset to reperfusion, and CTA collateral grade.

c Beta (95% CI).

## Discussion

Antiplatelet agents may be a promising adjunctive therapy during EVT for stroke. However, as antiplatelet agents are not administered systematically during EVT procedures, we evaluated safety and efficacy of prior antiplatelet therapy in a large observational study of patients treated with EVT for ischemic stroke in the Netherlands. In this study, we did not find an association between prior antiplatelet therapy and any of the safety outcomes. Particularly, the risk of sICH, nor the risk of death within 90 days was increased in patients on prior antiplatelet therapy. The associations with the secondary outcomes for efficacy were not significant. This concerned functional outcome at 90 days, stroke severity at 24 h assessed with the NIHSS, and post-EVT reperfusion grade (eTICI).

Our findings are consistent with the results of two smaller observational studies evaluating prior antiplatelet therapy in EVT-treated patients, both reporting no significant associations on risk of sICH and functional outcome.^18,19^ Our study negates the observation in the MR CLEAN trial of a substantially increased risk of sICH in patients on prior antiplatelet therapy.^20^

The safety of antiplatelet agents in patients treated with intravenous alteplase has been investigated in several studies. In the National Institute of Neurological Disorders and Stroke trial on the effect of intravenous alteplase, clinical deterioration was less common in patients who were on prior antiplatelet therapy.^21^ The authors suggested an association with early re-occlusion prevention. These findings formed the rationale for a trial of acetylsalicylic acid directly after intravenous alteplase treatment. This trial was halted prematurely because of futility and increased sICH risk.^4^ These results are not generalizable to patients undergoing EVT, because these patients will be more susceptible to re-occlusion and induction of microthrombi by vessel wall damage, and are therefore at higher risk of ischemic complications.

Given the liberal inclusion criteria of this registry and the broad area of common support in propensity scores after matching, we consider the results of this study are generalizable to the larger EVT-eligible population of ischemic stroke patients with an intracranial occlusion of the anterior circulation who are treated within 6.5 h from symptom onset.

Our study has some limitations. First, despite (I) propensity-score matching to obtain properly matched groups of patients with and without prior antiplatelet therapy and (II) additional covariate adjustment to increase robustness of the outcomes, it is still possible that our results are hampered by confounding indications. Factors relating to the patients vascular may not be captured completely in the model. Additionally, occurrence of sICH is impacted by several other factors such as the post (peri)procedural blood pressure management or follow-up infarct volume. These unmeasured characteristics may confound differences between patients with and without prior antiplatelet therapy. Second, re-occlusions were not scored systematically on follow-up imaging in this registry after EVT. Instead, we reported occurrence of stroke progression and new ischemic stroke, which showed a non-significant trend toward lower occurrence in patients who were on prior antiplatelet therapy. Third, in this study, we were not able to address the question whether dual antiplatelet therapy is associated with different safety and efficacy results as compared to single antiplatelet therapy due to low numbers, which warrants further study. Finally, the compliance with prior antiplatelet therapy in our cohort is unknown. If the compliance was poor, which is conceivable as antiplatelet therapy is paradoxically used in the prevention of stroke, this may have influenced our results.

The results of our observational study do not exclude the possibility of a sizeable beneficial effect of antiplatelet therapy in ischemic stroke patients undergoing EVT. In the ongoing MR CLEAN-MED trial (*Multicenter randomized clinical trial of endovascular treatment for acute ischemic stroke; the effect of periprocedural medication: acetylsalicylic acid, unfractionated heparin, both or neither*; ISRCTN76741621), patients are being randomized to intravenous acetylsalicylic acid and/or unfractionated heparin to investigate whether this will improve functional outcome after EVT. In this trial, intravenous acetylsalicylic acid is administered during EVT, overcoming the issue of non-compliance. This trial will provide new randomized data to answer the question whether platelet inhibition is safe and beneficial for this group of severely affected ischemic stroke patients.

## Conclusion

In this observational study, we did not find evidence that prior antiplatelet therapy is associated with sICH, functional outcome, reperfusion, or mortality after EVT for ischemic stroke. Our results do not exclude a beneficial or detrimental effect of antiplatelet therapy on outcome after EVT for ischemic stroke. A randomized trial is therefore justified to evaluate the safety and efficacy of antiplatelet agents administered during EVT.

## Supplemental Material

sj-pdf-1-wso-10.1177_1747493020946975 - Supplemental material for Prior antiplatelet therapy in patients undergoing endovascular treatment for acute ischemic stroke: Results from the MR CLEAN Registry Click here for additional data file. Supplemental material, sj-pdf-1-wso-10.1177_1747493020946975 for Prior antiplatelet therapy in patients undergoing endovascular treatment for acute ischemic stroke: Results from the MR CLEAN Registry by Rob A van de Graaf, Sanne M Zinkstok, Vicky Chalos, Robert-Jan B Goldhoorn, Charles BLM Majoie, Robert J van Oostenbrugge, Aad van der Lugt, Diederik WJ Dippel, Yvo BWEM Roos, Hester F Lingsma, Adriaan CGM van Es, Bob Roozenbeek and on behalf of the MR CLEAN Registry investigators in International Journal of Stroke
