# Supplementary material for: Prior antiplatelet therapy in patients undergoing endovascular treatment for acute ischemic stroke: Results from the MR CLEAN Registry
Source: Int J Stroke. 2020 Aug 14;16(4):476–85. doi: 10.1177/1747493020946975 (PMC8193619; doi:10.1177/1747493020946975)
Supplement: sj-pdf-1-wso-10.1177_1747493020946975 - Supplemental material for Prior antiplatelet therapy in patients undergoing endovascular treatment for acute ischemic stroke: Results from the MR CLEAN Registry [file sj-pdf-1-wso-10.1177_1747493020946975.pdf]

SUPPLEMENTAL MATERIAL ON:

PRIOR ANTIPLATELET THERAPY IN PATIENTS UNDERGOING ENDOVASCULAR TREATMENT FOR ACUTE ISCHEMIC STROKE: RESULTS FROM THE MR CLEAN REGISTRY

Rob A. van de Graaf, MD[1,2]; Sanne M. Zinkstok, MD, PhD[3]; Vicky Chalos, MD[1,2,4]; Robert-Jan B. Goldhoorn, MD[5]; Charles B.L.M. Majoie, MD, PhD[6]; Robert J. van Oostenbrugge, MD, PhD[5]; Aad van der Lugt, MD, PhD[2]; Diederik W.J. Dippel, MD, PhD[1]; Yvo B.W.E.M. Roos, MD, PhD[7]; Hester F. Lingsma, PhD[4]; Adriaan C.G.M. van Es, MD, PhD[2]; Bob Roozenbeek, MD, PhD[1,2]; on behalf of the MR CLEAN Registry Investigators*

Affiliations:

1. Department of Neurology, Erasmus MC University Medical Center, Rotterdam, The Netherlands
2. Department of Radiology & Nuclear Medicine, Erasmus MC University Medical Center, Rotterdam, The Netherlands
3. Department of Neurology, Tergooi, Hilversum, The Netherlands
4. Department of Public Health, Erasmus MC University Medical Center, Rotterdam, The Netherlands
5. Department of Neurology, Cardiovascular Research Institute Maastricht, Maastricht University Medical Center, Maastricht, The Netherlands
6. Department of Radiology and Nuclear Medicine, Amsterdam UMC, location AMC, Amsterdam, The Netherlands
7. Department of Neurology, Amsterdam UMC, University of Amsterdam, location AMC, Amsterdam, The Netherlands

Supplemental Material I

**Supplemental table.** Multiple imputation method	
Software	R 3.5.0, and the 'mice' package.
Imputed variables	*age, sex, baseline NIHSS score, glucose level, diabetes, thrombocyte count, previous myocardial infarction, previous stroke, hypercholesterolemia, hypertension, peripheral artery disease, atrial fibrillation, drug use (antiplatelet, coumarine, novel oral anticoagulant, statin and antihypertensive), prestroke mRS score, international normalized ratio, blood pressure, baseline ASPECTS, occlusion segment, affected hemisphere, collateral status, time from symptom onset to start of endovascular treatment, time from symptom onset to successful reperfusion or last contrast bolus, procedure duration, center, intravenous alteplase administration, iv heparin use, intra-arterial thrombolysis, general anesthesia performed, extended thrombolysis in cerebral infarction score at the beginning and end of the intervention, NIHSS score after 24-48 hours, new ischemic stroke, progression of stroke, extracranial hemorrhage, cardiac ischemia, sICH, functional outcome expressed on the modified Rankin Scale*
Number of imputed datasets	*5*
Seed	*41*

Supplemental Material II

A

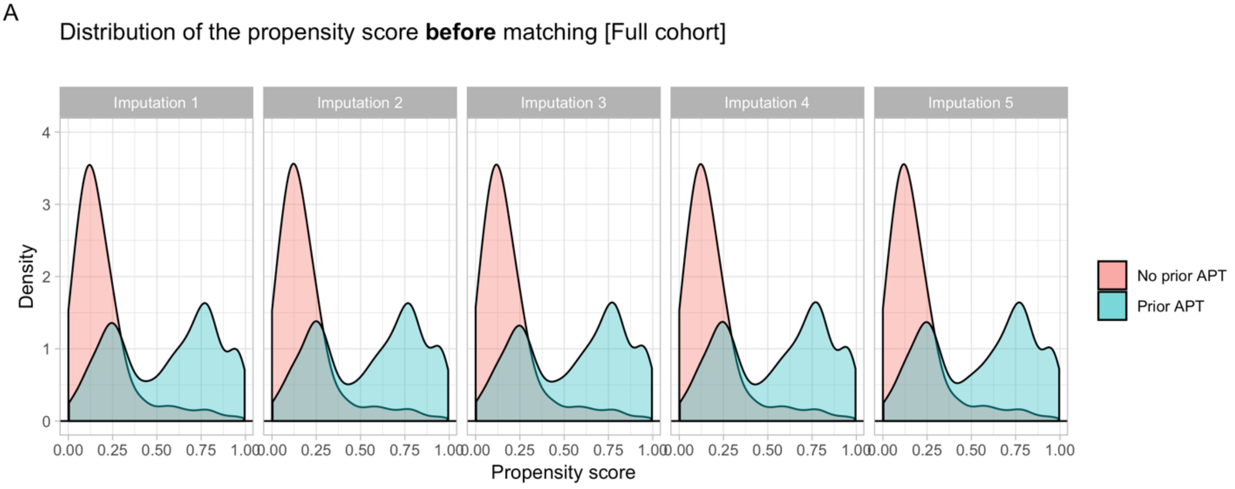

B

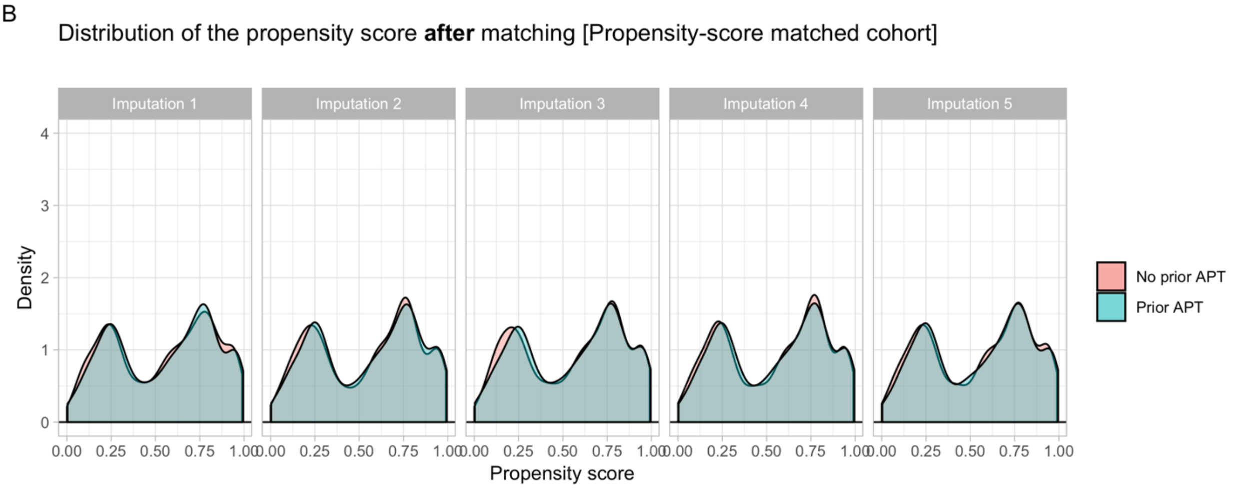

Supplemental Material III

Supplemental table. Primary and secondary outcomes in patients on prior antiplatelet therapy vs. no prior antiplatelet therapy in the full cohort using logistic and linear regression.

	Prior APT (n=937)	***No prior APT (n=2217)***	***(c)OR, (95%CI)***	***a(c)OR, (95%CI)*†**
Primary outcome				
Symptomatic intracranial hemorrhage	74 (7.9)	111 (5.0)	1.63 (1.20-2.21)	1.48 (0.99-2.20)
Secondary outcomes				
mRS at 90 days	4 [2, 6]	3 [2, 6]	0.70 (0.61-0.81)	0.92 (0.76-1.10)
mRS ≤ 2 at 90 days	312 (36)	882 (43)	0.74 (0.63-0.87)	1.00 (0.78-1.28)
NIHSS at 24-48 hours	10 [4, 17]	10 [4,17]	0.70 (0.01-1.39)[2]	-0.05 (-0.79 to 0.70)‡
Recanalization after intervention (eTICI ≥ 2B)	572 (63)	1321 (61)	1.06 (0.90-1.25)	1.07 (0.87-1.31)
Mortality at 90 days	309 (35)	540 (26)	1.53 (1.29-1.81)	1.27 (1.00-1.61)
Progression of stroke	71 (7.6)	211 (9.5)	0.78 (0.59-1.03)	0.81 (0.57-1.16)
New ischemic stroke	14 (1.5)	35 (1.6)	0.95 (0.51-1.77)	0.56 (0.24-1.29)
Extracranial hemorrhage	22 (2.3)	48 (2.2)	1.09 (0.65-1.81)	0.93 (0.49-1.73)
Cardiac ischemia	7 (0.7)	15 (0.7)	1.10 (0.45-2.72)	1.00 (0.33-3.07)

Summary: Primary and secondary outcomes of patients on prior antiplatelet therapy vs. no prior antiplatelet therapy. Skewed continuous and ordinal data are presented as median [IQR]. Binary data are presented as numbers (%).

Abbreviations: a(c)OR, adjusted (common) odds ratio; APT, antiplatelet therapy; CI, confidence interval; eTICI, extended thrombolysis in cerebral infarction; mRS, modified Rankin Scale

† *Variables in the multivariable logistic regression model:* age, sex, NIHSS at admission, pre-stroke mRS, intravenous alteplase, pre-interventional eTICI score, direct oral anticoagulant therapy, vitamin K antagonist therapy, previous stroke, myocardial infarction, peripheral artery disease, hypertension, atrial fibrillation, diabetes mellitus, intra-arterial thrombolysis, glucose at baseline, systolic blood pressure, anesthesia type (GA vs NGA), occlusion segment, ASPECTS at baseline, international normalized ratio, onset to recanalization, CTA collateral grade, heparin therapy

‡ Beta (95%CI)

Supplemental Material IV

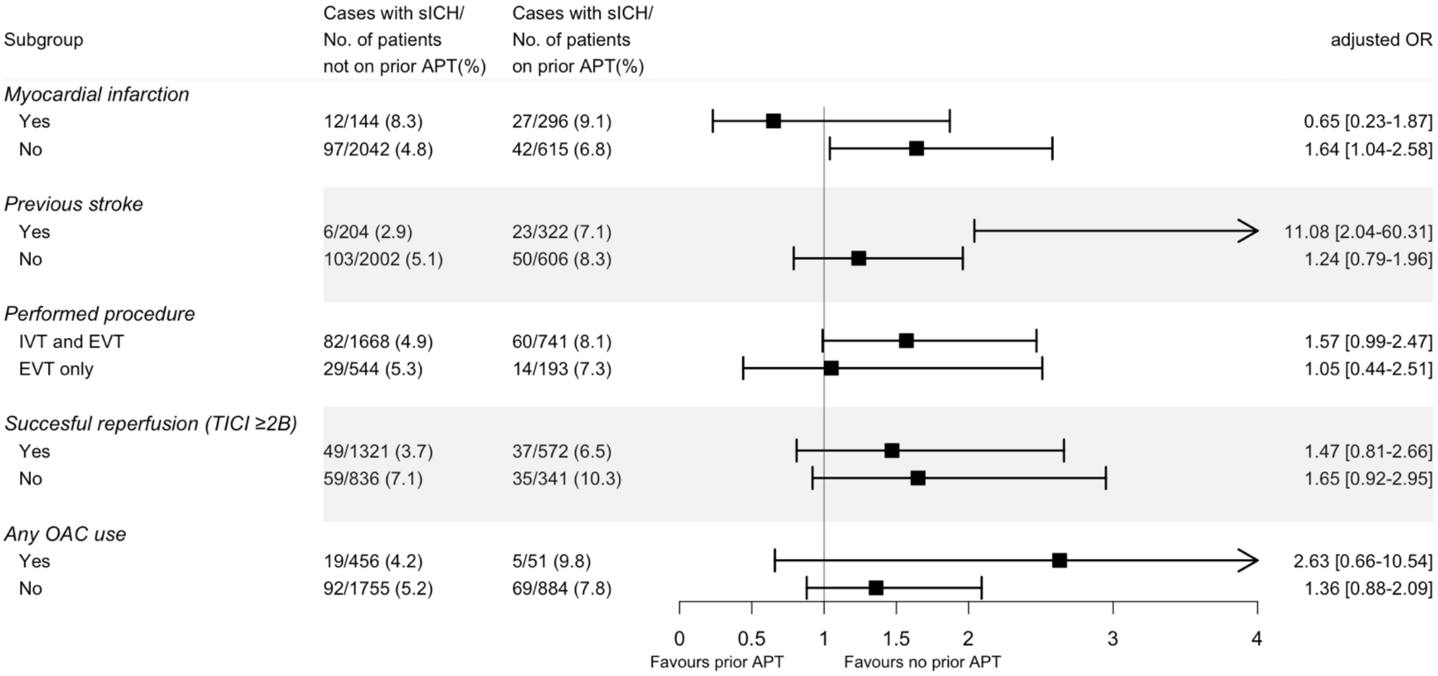

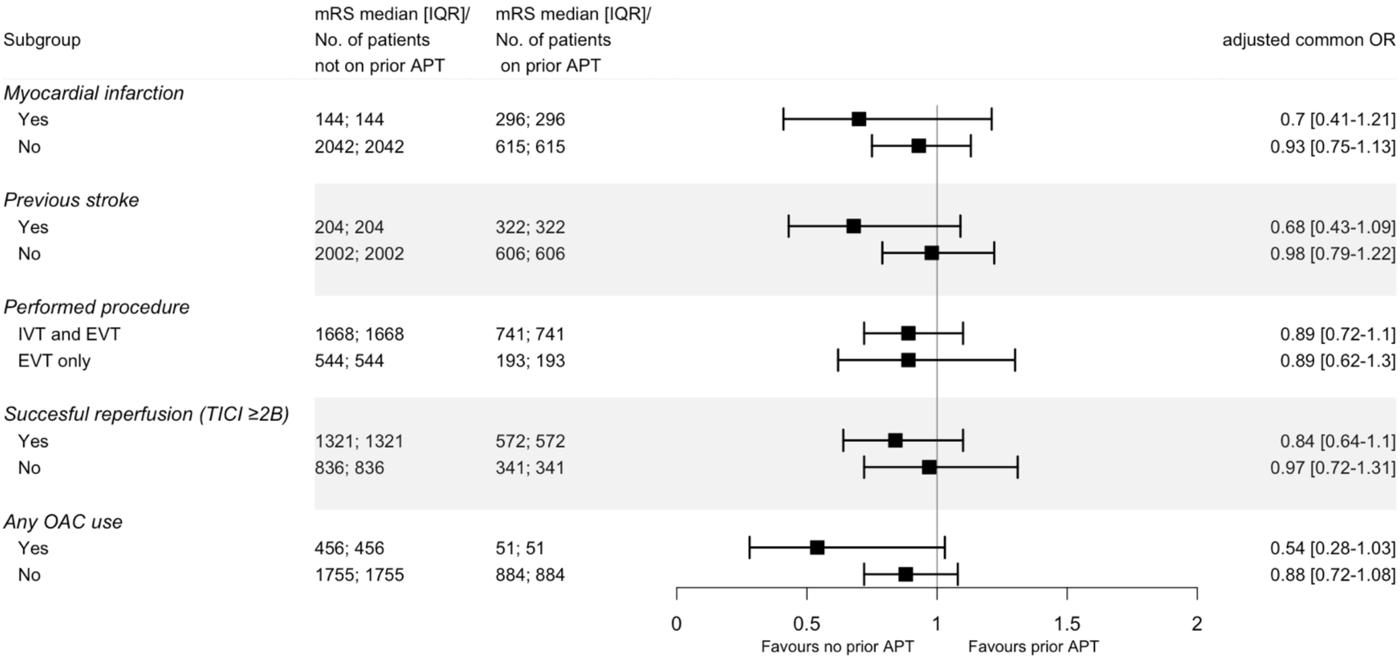

Supplemental material V

MR CLEAN Registry Investigators – group authors

Executive committee

Diederik W.J. Dippel[1];Aad van der Lugt[2];Charles B.L.M. Majoie[3];Yvo B.W.E.M. Roos[4];Robert J. van Oostenbrugge[5];Wim H. van Zwam[6];Jelis Boiten[14];Jan Albert Vos[8]

Study coordinators

Josje Brouwer[4];Sanne J. den Hartog[1,2,40];Wouter H. Hinsenveld[5,6];Manon Kappelhof[3];Kars C.J. Compagne[2];Robert-Jan B. Goldhoorn[5,6]; Maxim J.H.L. Mulder[1,2];Ivo G.H. Jansen[3]

Local principal investigators

Diederik W.J. Dippel[1];Bob Roozenbeek[1];Aad van der Lugt[2];Adriaan C.G.M. van Es[2];Charles B.L.M. Majoie[3];Yvo B.W.E.M. Roos[4];Bart J. Emmer[3];Jonathan M. Coutinho[4];Wouter J. Schonewille[7];Jan Albert Vos[8]; Marieke J.H. Wermer[9];Marianne A.A. van Walderveen[10];Julie Staals[5];Robert J. van Oostenbrugge[5];Wim H. van Zwam[6];Jeannette Hofmeijer[11];Jasper M. Martens[12];Geert J. Lycklama à Nijeholt[13];Jelis Boiten[14];Sebastiaan F. de Bruijn[15];Lukas C. van Dijk[16];H. Bart van der Worp[17];Rob H. Lo[18];Ewoud J. van Dijk[19];Hieronymus D. Boogaarts[20];J. de Vries[22];Paul L.M. de Kort[21]; Julia van Tuijl[21] ; Jo P. Peluso[26];Puck Fransen[22];Jan S.P. van den Berg[22];Boudewijn A.A.M. van Hasselt[23];Leo A.M. Aerden[24];René J. Dallinga[25];Maarten Uyttenboogaart[28];Omid Eschgi[29];Reinoud P.H. Bokkers[29];Tobien H.C.M.L. Schreuder[30];Roel J.J. Heijboer[31];Koos Keizer[32];Lonneke S.F. Yo[33];Heleen M. den Hertog[22];Emiel J.C. Sturm[35]; Paul Brouwers[34]

Imaging assessment committee

Charles B.L.M. Majoie[3](chair);Wim H. van Zwam[6];Aad van der Lugt[2];Geert J. Lycklama à Nijeholt[13];Marianne A.A. van Walderveen[10];Marieke E.S. Sprengers[3];Sjoerd F.M. Jenniskens[27];René van den Berg[3];Albert J. Yoo[38];Ludo F.M. Beenen[3];Alida A. Postma[6];Stefan D. Roosendaal[3];Bas F.W. van der Kallen[13];Ido R. van den Wijngaard[13];Adriaan C.G.M. van Es[2];Bart J. Emmer[3];Jasper M. Martens[12]; Lonneke S.F. Yo[33];Jan Albert Vos[8]; Joost Bot[36], Pieter-Jan van Doormaal[2]; Anton Meijer[27];Elyas Ghariq[13]; Reinoud P.H. Bokkers[29];Marc P. van Proosdij[37];G. Menno Krietemeijer[33];Jo P. Peluso[26];Hieronymus D. Boogaarts[20];Rob Lo[18];Dick Gerrits[35];Wouter Dinkelaar[2]Auke P.A. Appelman[29];Bas Hammer[16];Sjoert Pegge[27];Anouk van der Hoorn[29];Saman Vinke[20].

Writing committee

Diederik W.J. Dippel[1](chair);Aad van der Lugt[2];Charles B.L.M. Majoie[3];Yvo B.W.E.M. Roos[4];Robert J. van Oostenbrugge[5];Wim H. van Zwam[6];Geert J. Lycklama à Nijeholt[13];Jelis Boiten[14];Jan Albert Vos[8];Wouter J. Schonewille[7];Jeannette Hofmeijer[11];Jasper M. Martens[12];H. Bart van der Worp[17];Rob H. Lo[18]

Adverse event committee

Robert J. van Oostenbrugge[5](chair);Jeannette Hofmeijer[11];H. Zwenneke Flach[23]

Trial methodologist

Hester F. Lingsma[40]

Research nurses / local trial coordinators

Naziha el Ghannouti[1];Martin Sterrenberg[1];Corina Puppels[7];Wilma Pellikaan[7];Rita Sprengers[4];Marjan Elfrink[11];Michelle Simons[11];Marjolein Vossers[12];Joke de Meris[14];Tamara Vermeulen[14];Annet Geerlings[19];Gina van Vemde[22];Tiny Simons[30];Cathelijn van Rijswijk[21];Gert Messchendorp[28];Nynke Nicolaij[28];Hester Bongenaar[32];Karin Bodde[24];Sandra Kleijn[34];Jasmijn Lodico[34]; Hanneke Droste[34];Maureen Wollaert[5];Sabrina Verheesen[5];D. Jeurrissen[5];Erna Bos[9];Yvonne Drabbe[15];Michelle Sandiman[15];Marjan Elfrink[11];Nicoline Aaldering[11];Berber Zweedijk[17];Mostafa Khalilzada[15];Jocova Vervoort[21];Hanneke Droste[34];Nynke Nicolaij[2];Michelle Simons[11];Eva Ponjee[22];Sharon Romviel[19];Karin Kanselaar[19];Erna Bos[9];Denn Barning[10].

PhD / Medical students:

Esmee Venema[40]; Vicky Chalos[1,40];Ralph R. Geuskens[3]; Tim van Straaten[19];Saliha Ergezen[1]; Roger R.M. Harmsma[1]; Daan Muijres[1]; Anouk de Jong[1];Olvert A. Berkhemer[1,3,6];Anna M.M. Boers[3,39]; J. Huguet[3];P.F.C. Groot[3];Marieke A. Mens[3];Katinka R. van Kranendonk[3];Kilian M. Treurniet[3];Ivo G.H. Jansen[3];Manon L. Tolhuisen[3,39];Heitor Alves[3];Annick J. Weterings[3],Eleonora L.F. Kirkels[3],Eva J.H.F. Voogd[11];Lieve M. Schupp[3];Sabine Collette[28,29];Adrien E.D. Groot[4];Natalie E. LeCouffe[4];Praneeta R. Konduri[39];Haryadi Prasetya[39];Nerea Arrarte-Terreros[39];Lucas A. Ramos[39].

List of affiliations

Department of Neurology[1], Radiology[2], Public Health[40], Erasmus MC University Medical Center;
Department of Radiology and Nuclear Medicine[3], Neurology[4], Biomedical Engineering & Physics[39], Amsterdam UMC, University of Amsterdam, Amsterdam;
Department of Neurology[5], Radiology[6], Maastricht University Medical Center and Cardiovascular Research Institute Maastricht (CARIM);
Department of Neurology[7], Radiology[8], Sint Antonius Hospital, Nieuwegein;
Department of Neurology[9], Radiology[10], Leiden University Medical Center;
Department of Neurology[11], Radiology[12], Rijnstate Hospital, Arnhem;
Department of Radiology[13], Neurology[14], Haaglanden MC, the Hague;
Department of Neurology[15], Radiology[16], HAGA Hospital, the Hague;
Department of Neurology[17], Radiology[18], University Medical Center Utrecht;
Department of Neurology[19], Neurosurgery[20], Radiology[27], Radboud University Medical Center, Nijmegen;
Department of Neurology[21], Radiology[26], Elisabeth-TweeSteden ziekenhuis, Tilburg;
Department of Neurology[22], Radiology[23], Isala Klinieken, Zwolle;
Department of Neurology[24], Radiology[25], Reinier de Graaf Gasthuis, Delft;
Department of Neurology[28], Radiology[29], University Medical Center Groningen;
Department of Neurology[30], Radiology[31], Atrium Medical Center, Heerlen;
Department of Neurology[32], Radiology[33], Catharina Hospital, Eindhoven;
Department of Neurology[34], Radiology[35], Medical Spectrum Twente, Enschede;

Department of Radiology[36], Amsterdam UMC, Vrije Universiteit van Amsterdam, Amsterdam;
Department of Radiology[37], Noordwest Ziekenhuisgroep, Alkmaar;
Department of Radiology[38], Texas Stroke Institute, Texas, United States of America.
